# Waterpipe Smoking Among Japanese Teens and Young Adults: an Emerging Public Health Concern

**DOI:** 10.1093/ntr/ntaf252

**Published:** 2026-01-28

**Authors:** Sora Yamasaki, Aya Goto, Chihaya Koriyama, Takahiro Tabuchi

**Affiliations:** Tohoku University School of Medicine, Sendai, Miyagi, Japan; Department of Global Health and Population, Harvard T.H. Chan School of Public Health, Boston, MA, USA; Center for Integrated Sciences and Humanities, Fukushima Medical University, Fukushima City, Fukushima, Japan; Department of Epidemiology and Preventive Medicine, Kagoshima University Graduate School of Medical and Dental Sciences, Kagoshima City, Kagoshima, Japan; Division of Epidemiology, School of Public Health, Tohoku University Graduate School of Medicine, Sendai, Miyagi, Japan

## Abstract

**Introduction:**

Waterpipe (shisha) smoking has gained popularity among teens and young adults and is now prevalent in Japan. Yet, waterpipe smoking in the Japanese population remains understudied. This study examined the prevalence, characteristics, and knowledge/attitudes related to waterpipe use among Japanese teens and young adults, aiming to inform efforts to prevent further use.

**Methods:**

A cross-sectional analysis of the 2024 Japan Society and New Tobacco Internet Survey was conducted among individuals aged 16–29 years (N = 5564). Inverse probability weighting was used to generate estimates representative of the Japanese national population. Descriptive analyses assessed waterpipe use prevalence, use status, and reasons for use. Multivariable logistic regression—controlling for demographic, socioeconomic, and substance use covariates—evaluated associations between knowledge/attitudes and any lifetime (versus never) waterpipe use.

**Results:**

Among teens and young adults, 10.2% reported waterpipe use, with 65% of current users smoking occasionally. Usage was highest among the 20–24 age group. Common reasons for use included peer influence (59.7%), interest in shisha bars/cafés (54.2%) or interest in flavors (47.9%), and perceived reduced harm (40.7%). After adjusting for covariates, perceiving waterpipe use as less harmful (adjusted odds ratio [AOR] = 1.75) or “cool” (AOR = 1.44) remained strongly associated with significantly higher odds of lifetime use.

**Conclusions:**

Waterpipe smoking prevalence among young people in Japan is a public health concern, driven by peer influence, product appeal, and misperceptions of reduced harm. Targeted education and regulation to address perceptions about harm and social acceptability, along with continued monitoring, are warranted.

**Implications:**

To our knowledge, this is the first study to examine waterpipe smoking among teens and young adults in Japan, the most prevalent age group for waterpipe smokers globally. The reasons cited for use were similar to those in other countries, including peer influence and appealing flavors, with misconceptions about reduced harm and viewing waterpipe use as “cool” being particularly associated with use. Efforts to address misperceptions of reduced harm and social desirability are essential to reducing youth vulnerability. Our findings highlight the need for continued monitoring, targeted education, and policy interventions to tackle this emerging public health concern.

## Introduction

Waterpipe smoking (also known as shisha) has been gaining popularity worldwide, raising significant global health concerns. Originating from the Middle East and developing regions, its use has steadily expanded over the past two decades to North America, Europe, and Asia, with the number of waterpipe smokers increasing each year.^1^^,^^2^ Typical waterpipe users are younger, male, urban residents with relatively high education and income levels.^1^^,^^3^^,^^4^ This global trend has now reached Japan, where waterpipe smoking among young adults is rapidly increasing.^5^ Although official statistics are unavailable, according to Japan’s largest online directory and review site for shisha cafés, ~2000 shisha cafés or bars were operating nationwide as of March 2025, which has doubled since the end of 2023.^6^^,^^7^

Waterpipe smoking involves a device in which charcoal-heated air passes through flavored tobacco, creating smoke that travels through water at the base of the instrument before being inhaled by the smoker through a mouthpiece.^1^^,^^8^^,^^9^ Users typically visit shisha cafés or bars to use waterpipes either alone or socially in groups, often sharing one pipe with separate mouthpieces over sessions lasting up to 180 minutes.^10^ In addition to this social aspect, the erroneous belief that the water filters out toxins also makes waterpipes more appealing to teens and young adults than other tobacco products.^1^^,^^11^^,^^12^ Another key driver behind the global popularity of waterpipe smoking is the pleasant, sweet flavors, such as apple or mint.^13^ Additionally, many users believe that waterpipe smoking is less harmful and addictive than cigarettes and often report a sense of relaxation.^14^ However, waterpipe smoke contains a range of toxic substances, including nicotine and carcinogens, with some substances such as carbon monoxide often found at higher levels than in cigarette smoke.^15^ Several adverse health effects have also been reported.^16^^,^^17^

In addition to its direct health effects, waterpipe smoking may amplify overall health risks by serving as a gateway to other tobacco products. One study in the United States found that 35% of waterpipe smokers who were non-cigarette smokers reported intentions to start cigarette smoking within the following year.^18^ In Japan, it is essential to acknowledge the frequent use of heated tobacco products (HTPs) when understanding the role and trajectory of waterpipe use, given the dominance of HTPs within the country’s novel tobacco product market.^19^

Although waterpipe smoking among teens and young adults is increasing globally, research on this issue remains scarce in the Western Pacific region, including Japan.^8^ A recently published study on waterpipe use across the entire Japanese population found that the 20–29 age group had the highest prevalence.^5^ Building on this finding, our study provides a more focused and in-depth analysis of teens and young adults. We aimed to move beyond prevalence by investigating the specific reasons for use, knowledge, and attitudes associated with waterpipe smoking among this population. Additionally, to provide valuable insights into Japan’s unique tobacco landscape, we compared these waterpipe behaviors with those observed for HTPs. These findings will help inform targeted interventions and evidence-based tobacco control policies.

## Methods

### Study Design and Subjects

This study analyzed data from the 2024 Japan Society and New Tobacco Internet Survey (JASTIS 2024; https://jastis-study.jp), a cross-sectional, web-based survey conducted from January 24 to February 27, 2024, by a Japanese internet research agency (Rakuten Insight, Inc. https://insight.rakuten.co.jp/). JASTIS is an ongoing longitudinal internet research project to examine the current usage, regulatory context, and health effects of new tobacco products in Japan.^20^ In 2024, the survey specifically included questions assessing knowledge and attitudes toward waterpipe use. Participants were randomly selected from Rakuten Insight’s extensive online panel, comprising several million users reflecting diverse demographic characteristics of the general Japanese population (eg education, marital status, housing tenure). Initial sampling was stratified by sex, age, and prefecture to represent all 47 Japanese prefectures based on the 2019 population distribution, and recruitment continued until the target sample size was achieved.

To maintain data validity, responses were carefully screened using predefined quality-control criteria. Specifically, responses were excluded if they showed inconsistencies or indications of low-quality data, such as excessively short response times (less than 10 minutes), improbable demographic answers (eg excessively large household sizes), incorrect answers on attention-check questions, or logically inconsistent reporting. Of the initial 32 000 respondents (aged 16–83), 2732 were excluded on the basis of these rigorous quality-control criteria, resulting in a final analytic sample of 29 268 participants.

For this analysis, a subset of teens and young adults with 5564 participants aged 16–29 years was selected, considering both the prevalence of waterpipe smoking in this age group (Table 1) and the Japanese policy definitions of young adults outlined in the *Kodomo Kihon Hou* (Child Basic Law) and related 2023 guidelines.^21^

**Table 1:** Weighted Prevalence of Any Lifetime Waterpipe Use by Age Group

	n/N (%)^a^		
		Sex	
Age	Total (N = 29 268)	Male (N = 14 453)	Female (N = 14 817)
16–19	23/521 (4.3)	12/158 (7.9)	10/363 (2.8)
20–24	197/1759 (11.2)	105/762 (13.7)	92/997 (9.3)
25–29	348/3284 (10.6)	229/1722 (13.3)	119/1562 (7.6)
30–34	194/2069 (9.4)	141/955 (14.8)	53/1114 (4.8)
35–39	203/2756 (7.4)	134/1402 (9.6)	69/1354 (5.1)
40–44	154/2387 (6.4)	111/1197 (9.2)	43/1189 (3.6)
45–49	106/2650 (4.0)	75/1337 (5.6)	30/1314 (2.3)
50–54	66/2344 (2.8)	54/1203 (4.5)	12/1141 (1.1)
55–59	64/2277 (2.8)	48/1142 (4.3)	15/1135 (1.3)
60–64	38/2247 (1.7)	31/1145 (2.7)	7/1102 (0.6)
65–69	55/2238 (2.5)	52/1086 (4.8)	3/1152 (0.2)
70+	44/4736 (0.9)	37/2344 (1.6)	6/2394 (0.3)

a. Weighted to be nationally representative based on the 2019 Comprehensive Survey of Living Conditions. n/N (%): n = number of waterpipe users; N = total number of individuals in the group; % = proportion of any lifetime waterpipe use within that group.

### Variables

Waterpipe use was assessed through self-report and categorized into four groups. Participants who indicated never using a waterpipe in their lifetime were classified as “Never” users. Those who reported lifetime use but no use in the past 30 days were classified as “Ever” users. Among past-30-days users, individuals who reported 1–3 days of use within the past 30 days were classified as “Current Occasional” users, and those who reported use on four or more days within the past 30 days, corresponding to at least weekly use, were classified as “Current Regular” users.^18^^,^^22^ For logistic regression analyses, participants were dichotomized based on any lifetime waterpipe use (combining ever, current occasional, and current regular users) versus no lifetime waterpipe use (never users).

The use of HTPs, which entered the market in 2014 and are commonly used in Japan today, was also assessed and categorized using the same four groups (“Never,” “Ever,” “Current Occasional,” and “Current Regular”). Like waterpipe use, these categories were dichotomized in relevant logistic regression analyses to compare any lifetime HTP use (combining ever, current occasional, and current regular users) against no lifetime HTP use (never users).

Several potential covariates were controlled for in multivariable analyses. Demographic variables included age (continuous) and sex (male, female). Densely inhabited district classification (metropolitan area, large city, accessible small town, remote small town, accessible rural settlement, remote rural settlement) was used for geographic location.^5^^,^^23^ Socioeconomic factors included education level (high school/technical school, college/university or above) and self-reported equivalent household income (calculated using a simplified OECD-modified equivalence scale categorized into quartiles). Conventional cigarette smoking was defined as any reported use of pre-rolled or hand-rolled cigarettes. Additional substance use was assessed through alcohol consumption (categorized as no, light, moderate, or heavy drinking based on frequency and quantity) and illicit drug use (categorized as yes or no).

Social network size was assessed with the Lubben Social Network Scale (LSNS-6),^24^ specifically its Family and Friends subscales. Each subscale comprises three items rated on a six-point scale evaluating social relationships, yielding scores ranging from 0 to 15 (higher scores indicate stronger social connectivity). Waterpipe-related knowledge (harm to smokers and others), attitudes (perceived “coolness” of waterpipe smoking; ie its perceived social appeal), and reasons for waterpipe use were assessed using a four-point scale (“Agree,” “Somewhat agree,” “Somewhat disagree,” “Disagree”). The list of reasons for waterpipe use was adapted from Japanese studies on HTPs,^25^ which were informed by prior research on e-cigarette motivations.^26^ The item “interest in shisha bars/cafés” was added to specifically address the waterpipe context. This was subsequently dichotomized into yes (combining “Agree” and “Somewhat agree”) and no (combining “Somewhat disagree” and “Disagree”).

### Statistical Analysis

To assess sample representativeness, we compared demographic characteristics across our unweighted sample, weighted sample, and 2020 National Census data^27^ (Supplementary Table S1). For the weighted analysis, we employed inverse probability weighting to enhance Japanese national representativeness of our internet-based sample. Propensity scores were estimated via logistic regression using data from our sample and a probability sample from the 2019 Comprehensive Survey of Living Conditions.^28^ The weighting procedure adjusted for differences in sex, age, smoking status, educational status, housing tenure, marital status, geographic region, and subjective health status.^20^ All main results presented use weighted data to ensure national representativeness, with corresponding unweighted results provided in the Supplementary Materials.

We first calculated descriptive statistics (frequencies and proportions) for waterpipe use prevalence and reported reasons for waterpipe use, focusing on individuals aged 16–29. Considering the distinct difference in reasons for waterpipe smoking reported outside Japan,^1^ sex-stratified differences in the distribution of original four-level responses were assessed by the Rao-Scott chi-squared test. Then, unadjusted logistic regression analyses were used to examine each covariate individually about any lifetime waterpipe use (versus no use). Finally, adjusted logistic regression analyses were conducted to assess associations between knowledge and attitudes relating to any lifetime waterpipe use, controlling for all covariates listed in Table 2. A significance level of *p* < .05 was considered statistically significant. To identify specific characteristics of waterpipe smoking, an analogous set of weighted descriptive and logistic regression analyses were also performed for HTP use for comparison, controlling for the same covariates as in the waterpipe analyses; these results are presented in the Supplementary Material.

**Table 2:** Weighted Comparison of Characteristics by Waterpipe Use Status Among Teens and Young Adults (Age 16–29)

Characteristics	N (%)^a^ or *Mean (SD)*^b^					Comparison^d^
	Total^c^	Never	Ever	Occasional	Regular	Crude OR (95% CI)
	(N = 5564)	4997 (89.8)	303 (5.4)	173 (3.1)	92 (1.7)	
Age	*24.71 (3.53)*	*24.69 (3.6)*	*25.27 (2.9)*	*24.23 (3.6)*	*24.95 (2.7)*	1.02 (0.99–1.04)
Sex						
Male	2642 (47.5)	2296 (86.9)	170 (6.4)	110 (4.2)	67 (2.5)	1
Female	2922 (52.5)	2701 (92.4)	133 (4.6)	63 (2.1)	25 (0.9)	0.54 (0.45–0.65)
Densely Inhabited District						
Metropolitan areas	2748 (49.4)	2469 (51.6)	156 (52.6)	82 (48.9)	41 (46.6)	1
Large cities	784 (14.1)	714 (14.9)	31 (10.4)	34 (20.5)	5 (5.2)	0.86 (0.65–1.14)
Accessible small towns	250 (4.5)	228 (4.8)	8 (2.6)	4 (2.4)	11 (12.1)	0.87 (0.55–1.36)
Remote small towns	306 (5.5)	252 (5.3)	24 (8.1)	19 (11.6)	11 (12.7)	1.92 (1.39–2.63)
Accessible rural settlements	658 (11.8)	592 (12.4)	35 (11.7)	16 (9.5)	16 (18.1)	0.99 (0.75–1.32)
Remote rural settlements	590 (10.6)	530 (11.1)	44 (14.7)	12 (7.2)	5 (5.4)	1.01 (0.75–1.35)
Equivalent Household Income						
Lowest	1545 (27.8)	1428 (28.6)	46 (15.2)	41 (24.0)	30 (32.1)	1
Lower-middle	1323 (23.8)	1186 (23.7)	83 (27.3)	41 (23.8)	13 (14.5)	1.41 (1.09–1.82)
Upper-middle	1549 (27.8)	1398 (28.0)	74 (24.6)	45 (26.1)	31 (34.1)	1.32 (1.02–1.69)
Highest	1147 (20.6)	985 (19.7)	100 (33.0)	45 (26.1)	18 (19.3)	2.02 (1.57–2.59)
Education Level						
High school, technical school	4030 (72.4)	3668 (73.4)	197 (65.0)	103 (59.7)	62 (67.3)	1
College, university, or above	1534 (27.6)	1328 (26.6)	106 (35.0)	70 (40.3)	30 (32.7)	1.57 (1.31–1.88)
Other Tobacco Product						
Conventional cigarette smokers	519 (9.3)	199 (4.0)	144 (47.7)	109 (63.3)	66 (72.3)	31.3 (25.1–38.9)
Conventional cigarette non-smokers	5045 (90.7)	4798 (96.0)	159 (52.4)	63 (36.7)	26 (27.7)	1
HTPs users	1222 (22.0)	764 (15.3)	235 (77.4)	136 (79.1)	87 (94.8)	23.2 (18.6–28.9)
HTPs non-users	4342 (78.0)	4233 (84.7)	69 (22.6)	36 (20.9)	5 (5.2)	1
Alcohol Consumption						
No drinking	2545 (45.8)	2454 (49.1)	54 (17.7)	32 (18.2)	6 (6.3)	1
Light drinking	2484 (44.6)	2158 (43.2)	167 (55.1)	101 (58.3)	58 (63.3)	4.07 (3.20–5.17)
Moderate drinking	485 (8.7)	345 (6.9)	77 (25.3)	38 (22.0)	25 (27.7)	10.9 (8.21–14.60)
Heavy drinking	50 (0.9)	39 (0.8)	6 (1.9)	3 (1.5)	3 (2.7)	7.48 (3.69–15.20)
Illicit Drug Usage						
No	5348 (96.1)	4912 (98.3)	256 (84.4)	119 (69.2)	61 (65.8)	1
Yes	216 (3.9)	84 (1.7)	47 (15.6)	53 (30.8)	31 (34.2)	17.70 (13.20–23.60)
Social Network						
Friends (range: 0–15)	*6.96 (3.86)*	*6.77 (3.9)*	*8.78 (3.6)*	*8.28 (2.8)*	*8.54 (3.0)*	1.13 (1.10–1.15)
Family (range: 0–15)	*7.92 (3.29)*	*7.91 (3.3)*	*8.10 (3.1)*	*8.03 (2.8)*	*7.97 (3.2)*	1.01 (0.99–1.04)

HTP: Heated Tobacco Product; OR: Odds ratio; CI: Confidence interval; SD: Standard deviation

Weighted to be nationally representative based on the 2019 Comprehensive Survey of Living Conditions.

a. Proportions are within each response group (row percentages).

b. Numbers in *italics* indicate continuous variables and represent mean (SD).

c. Column proportions are shown for the total distribution.

d. Logistic regression analysis was performed. Crude OR compares participants with no lifetime waterpipe use (never users, reference) against those with any lifetime waterpipe use (combining ever, occasional, and regular users).

All statistical analyses were performed with EZR (Saitama Medical Center, Jichi Medical University, Saitama, Japan), a graphical user interface for R (The R Foundation for Statistical Computing, Vienna, Austria). More precisely, EZR is a modified version of R commander designed to add statistical functions frequently used in biostatistics.^29^

### Ethical Consideration

This study was reviewed and approved by th*e Ethics Review Committee of the Osaka International Cancer Institute (No. 1611079163; November 7, 2016)* and the *Ethics Committee of the Tohoku University Graduate School of Medicine (No. 2024-1-231; June 27, 2024)*. All respondents provided informed consent, and they could discontinue or skip questions at any time.

## Results

**Figure 1:**
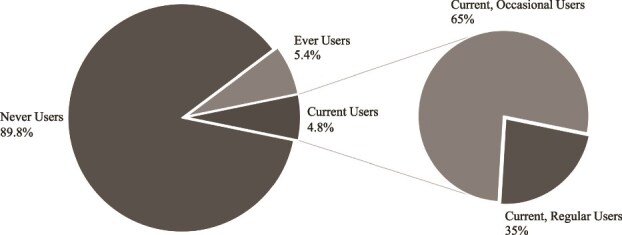
Waterpipe use status in teens and young adults (age 16–29, N = 5564).

Table 1 shows the weighted distribution of waterpipe use by age group of the total sample. In our sample, 747 teens and young adults (aged 16–29) reported any lifetime waterpipe use (Supplementary Table S2). After weighting, the highest prevalence was observed in the age group 20–24 (11.2%), followed by 25–29 (10.6%), and the prevalence decreased with increasing age. Figure 1 presents the detailed waterpipe use status among teens and young adults, where 10.2% reported lifetime use, and within current users, occasional use (65%) was the most common. The characteristics of the 5564 teens and young adult participants (mean age 24.7 years, 52.5% female) are detailed in Table 2, including the breakdown by waterpipe use status.

Participants reported various reasons for smoking waterpipes, which were grouped into three main categories: social influence, product appeal, and as a substitute for smoking cigarettes (hereafter, “substitution”) (Table 3). Overall, peer influence was the most frequently reported (59.7%), followed by interest in shisha bars/cafés (54.2%), interest in flavors (47.9%), and perceived as less harmful (40.7%). Significant differences were observed between sexes, and particularly for smoking substitution. For example, significantly more men cited “where other tobacco is restricted” as a reason than did women (45.5% vs 15.4%).

**Table 3:** Weighted Reasons for Smoking Waterpipe in Teens and Young Adults (Age 16–29)

	N (%)^a,b^		
Reason	Sex		Total
	Male (N = 347)	Female (N = 221)	(N = 568)
**Social Influence**			
Peer influence	206 (59.4)	133 (60.2)	339 (59.7)
Family influence	129 (37.2)^**^	49 (22.2)^**^	178 (31.3)
Seen as “cool”	159 (45.8)^*^	65 (29.4)^*^	224 (39.4)
**Product Appeal**			
Interest in flavors	169 (48.7)	103 (46.6)	272 (47.9)
Perceived less harmful	149 (42.9)	82 (37.1)	231 (40.7)
To avoid bothering others	128 (36.9)^*^	52 (23.5)^*^	180 (31.7)
Interest in shisha bars/cafés	203 (58.5)	105 (47.5)	308 (54.2)
**Smoking Substitution**			
Where other tobacco is restricted	158 (45.5)^***^	34 (15.4)^***^	192 (33.8)
To quit smoking	136 (39.2)^**^	45 (20.4)^**^	181 (31.9)
To reduce smoking	133 (38.3)^***^	39 (17.6)^***^	172 (30.3)

a. Weighted to be nationally representative based on the 2019 Comprehensive Survey of Living Conditions. Percentages represent column percentages of participants agreeing with the reason.

b. Significance based on Rao-Scott chi-squared test comparing subgroups (Male vs Female): ^*^p < .05, ^**^p < .01, ^***^p < .001.

Waterpipe-related knowledge and attitudes were also examined in relation to waterpipe use status (Table 4). Even after adjusting for demographic, socioeconomic, and other substance use covariates, perceptions of waterpipe use as not harmful to the smoker (adjusted odds ratio [AOR] = 1.75, 95% confidence interval [CI] 1.35–2.27), not harmful to others (AOR = 1.61, 95% CI 1.25–2.09), or as “cool” (AOR = 1.44, 95% CI 1.09–1.90) remained associated with significantly higher odds (*p* < .001) of being a waterpipe user compared with holding the opposite views.

**Table 4:** Weighted Knowledge and Attitudes Toward Waterpipe by User Status Among Teens and Young Adults (Age 16–29)

	N (%)			Comparison^a^	
	Total (N = 5564)	Never used waterpipe (N = 4997)	Used waterpipe (N = 567)	Crude OR (95% CI)	Adjusted OR (95% CI)
Waterpipes are harmful to users					
No	939 (16.9)	727 (18.9)	212 (39.7)	2.83 (2.34–3.42)	1.75 (1.35–2.27)
Yes	3450 (62.0)	3127 (81.1)	323 (60.3)	1	1
Waterpipes are harmful to others					
No	901 (16.2)	689 (17.9)	211 (40.1)	3.1 (2.53–3.72)	1.61 (1.25–2.09)
Yes	3471 (62.4)	3155 (82.1)	316 (59.9)	1	1
Waterpipes are “cool”					
No	3712 (66.7)	3365 (87.2)	347 (65.0)	1	1
Yes	682 (12.3)	495 (12.8)	187 (35.0)	3.7 (2.99–4.48)	1.44 (1.09–1.90)

OR: Odds ratio; CI: Confidence interval

Weighted to be nationally representative based on the 2019 Comprehensive Survey of Living Conditions.

a. Logistic regression analysis compares participants with any lifetime waterpipe use (combining ever, occasional, and regular users) against those with no lifetime use (never users, reference). Adjusted OR is controlled for covariates shown in Table 2.

Note: Responses of “Unaware of waterpipe”, excluded from OR analyses, constituted 17.7% (harmful to users), 18.2% (harmful to others), and 17.5% (“cool”) of the total sample.

Supplementary weighted analyses examined the use of HTPs and found its prevalence among teens and young adults in Japan to be 22.0% (N = 1223) (Supplementary Table S6). Among HTP users, the primary reasons were peer influence (59.1%), perceived reduced harm (43.7%), and to avoid bothering others (38.9%) (Supplementary Table S7). Perceptions that HTPs are not harmful to smokers (AOR = 2.15, 95% CI 1.70–2.72), are not harmful to others (AOR = 1.79, 95% CI 1.42 – 2.25), or are “cool” (AOR = 4.49, 95% CI 3.46–5.82) remained significant predictors of HTP use after covariate adjustment (Supplementary Table S8).

## Discussion

To the best of our knowledge, this was the first study to examine the prevalence and characteristics of waterpipe smokers among teens and young adults (aged 16–29 years) in Japan, utilizing a relatively large sample of 5564 teens and young adults. Although the 16–19 age group had fewer waterpipe smokers, this group was included to comprehensively represent current trends among teens and young adults. Recognizing that internet surveys often have sampling biases, we applied inverse probability weighting to more closely reflect the national population structure (Supplementary Table S1). Analysis of our sample showed that 10.2% have used a waterpipe; among current users, 65% were occasional users. Globally, “ever” use of waterpipes (ie having used them at least once) among this generation varies widely, from around 13% to 65% in the Eastern Mediterranean and around 3% to 50% for Western countries as of 2018.^8^ This suggests that Japanese waterpipe smokers now also represent a non-negligible proportion. Additionally, a U.S. study of college students reported that 6% of current users smoked daily or almost daily.^30^ Overall, our finding aligns with global trends, where the majority of users are occasional users, suggesting that waterpipe smoking is predominantly a social activity rather than a daily habit like other tobacco products.^1^^,^^30^

Additionally, our analysis revealed key motivations for waterpipe use among Japanese teens and young adults. Peer influence (59.7%) was the most prominent reason cited overall, followed by interest in shisha bars/cafés (54.2%), interest in flavors (47.9%), and perceived reduced harm (40.7%). These motivations reflect broader global trends, with a previous review study identifying four key drivers of waterpipe spread: (1) the introduction of flavored tobacco, which commercialized the product and made it more appealing, especially to young people; (2) the integration of waterpipe smoking with social café culture; (3) positive portrayal on the internet and on social media, with 92% of waterpipe-related videos showing smoking in a positive light; and (4) the lack of waterpipe-specific policies and regulations.^1^ These findings align with a systematic review examining motivations among students in Middle Eastern and Western countries, which similarly identified socialization, relaxation, pleasure, peer influence, and curiosity as primary factors.^31^ Furthermore, perceptions strongly predicted use. After adjusting for covariates, believing waterpipes to be less harmful to the user (AOR = 1.75) or “cool” (AOR = 1.44) significantly increased the odds of lifetime use. This aligns with a previous study conducted in Iraq among university students, where waterpipe smoking was widely perceived as less harmful or “cool” among users but not among non-users.^32^ However, despite its perceived social appeal, several studies highlight serious health risks, including respiratory and cardiovascular effects, and a significantly increased risk of oral cancer.^16^^,^^17^ Effective interventions are needed to bridge the gap between the perceptions and adverse health impacts of waterpipe use.

Interestingly, significant differences were noted between sexes in the reasons reported for using waterpipes. Specifically, men more frequently cited smoking substitution motives. This suggests potentially distinct motivations, with men more likely viewing waterpipe as an alternative to cigarettes. Of note, associations of knowledge and attitudes with waterpipe use did not significantly differ between sexes among users (data not shown). This might indicate homogeneous views on product risk and appeal, regardless of gender-specific motivations. Given these findings, interventions prioritizing behavioral approaches and awareness campaigns to correct misconceptions about harm and social appeal should be central, potentially incorporating sex-tailored messages addressing peer influence, substitution motives, and flavor appeal.

Comparing these waterpipe findings with supplementary analyses of HTP users revealed common and different primary motivations. Although peer influence (waterpipe 59.7%; HTPs 59.1%) and reduced harm perceptions (waterpipe 40.7%; HTPs 43.7%) were common reasons for both, seeing use as “cool” (waterpipe 39.4%; HTPs 30.4%) was cited more frequently by waterpipe users than by HTPs users. This pattern indicates that social appeal may be more central for waterpipe use than for HTPs. Furthermore, perceiving the products to be less harmful to the smoker (waterpipe AOR = 1.75; HTPs AOR = 2.15) or less harmful to others (waterpipe AOR = 1.61; HTPs AOR = 1.79) remained a significant predictor of their use, highlighting a shared vulnerability toward novel tobacco products among teens and young adults. Thus, interventions should comprehensively target harm misperceptions across a broader spectrum of novel tobacco products to effectively reduce youth vulnerability worldwide.

This study has several limitations. First, reliance on self-reported data introduces the potential for reporting errors. This was suggested by some inconsistencies where respondents reporting waterpipe or HTP use indicated unawareness of the product, but in a small fraction (1.7% to 3.5%) of respondents for waterpipe or HTP use. Such discrepancies, likely due to respondent errors such as misunderstanding or inattention, may lead to misclassification of prevalence and usage data. However, given the large sample size, this noise is unlikely to invalidate the primary prevalence estimates or strong associations observed. Also, while self-reporting can be subject to social desirability bias, several factors likely minimized this specific concern for usage reporting in our study. Participants were assured of confidentiality and explicitly informed that reporting illicit behaviors would not lead to legal consequences. Moreover, as the legal age for tobacco use in Japan is 20 and 96% of participants were above this age, intentional underreporting of smoking status itself due to perceived social undesirability was likely minimal. Second, the questionnaire’s closed-ended format for assessing motivations for waterpipe use may have limited participants’ ability to report other relevant factors. Future research should incorporate qualitative methods to explore potentially overlooked factors and to examine young people’s attitudes toward waterpipe use in greater depth. Third, this analysis was based solely on cross-sectional data from the 2024 JASTIS survey, limiting our ability to observe trends or establish causality. Therefore, continuous monitoring of waterpipe smoking trends in Japan is needed. Lastly, quantifying the amount of waterpipe smoke exposure is inherently difficult owing to unique use patterns, such as session length variability, sharing practices, and often intermittent use, unlike the more standardized consumption of cigarettes. Consequently, a validated standard metric to capture dose is lacking, and this study relied on self-reported usage frequency categories, which may not fully capture exposure intensity.^33^

Given our findings, there is an urgent need for tailored interventions to address misconceptions about waterpipes, particularly among teens and young adults. Previous research suggests that behavioral interventions in schools or community settings—such as sessions providing health information, correcting misunderstandings, and teaching refusal skills—can effectively improve young people’s knowledge, attitudes, and beliefs about waterpipe smoking.^34^ Furthermore, public information campaigns play a vital role not only by communicating clear information on health risks, but also by exposing tobacco industry tactics that target young people—an approach that has successfully reduced tobacco acceptance among adolescents.^35^ Regulatory measures such as restricting flavored tobacco products may also prove effective. For example, the Tobacco Control Act by the U.S. Food and Drug Administration, which banned cigarettes with characterizing flavors, resulted in a notable decrease in youth smoking rates.^36^ Additionally, taxation or price increases on waterpipe products could significantly reduce youth initiation, given the price sensitivity of teens and young adults.^34^^,^^35^ At present, a waterpipe session typically costs around 3000 yen (~20 USD), a price point that may be relatively accessible for this age group, especially when shared among peers.^6^ Finally, policies explicitly prohibiting youth-oriented marketing, particularly through social media and streaming platforms, could reduce exposure to marketing strategies that reinforce the attractiveness of waterpipe smoking.^35^

Globally, even countries with strict cigarette smoking regulations often lack equivalent policies for waterpipe smoking.^37^ Given the novelty of waterpipe smoking in Japan, educational campaigns and interventions remain even more limited. Google Trends data (www.google.com/trends) show a notable rise in searches for “shisha” and “shisha bar” in Japan starting around 2020, which has remained high through 2025, proving the recent increase in interest in waterpipes. However, one of the few examples of public health initiatives on waterpipe smoking in Japan is a brief mention of waterpipe-related health risks in a brochure on new tobacco products (e-cigarettes and HTPs) published by the Japan Medical Association.^38^ As a country with a unique tobacco landscape with a high prevalence of HTPs, further research on waterpipe smoking in Japan among younger generations can provide critical insights for other countries in addressing the growing popularity of waterpipe smoking. Thus, continued monitoring and future studies focused on developing comprehensive educational efforts and tailored regulatory policies must be implemented as a priority.

## Supplementary Material

Supplementary_material_ntaf252

## Data Availability

The data supporting this study contain potentially sensitive participant information and are not publicly available. Anonymized data are available from Dr. Tabuchi upon reasonable request by qualified researchers.
