# Supplementary material for: Waterpipe Smoking Among Japanese Teens and Young Adults: an Emerging Public Health Concern
Source: Nicotine Tob Res. 2026 Jan 28;28(7):1148–55. doi: 10.1093/ntr/ntaf252 (PMC13286634; doi:10.1093/ntr/ntaf252)
Supplement: Supplementary_material_ntaf252 [file supplementary_material_ntaf252.docx]

Supplementary Table S1. Comparison of Sample Characteristics with 2020 Japanese National Census Data for Teens and Young Adults (Age 16–29)

| Characteristic | Unweighted | | | Weighted^a^ | |  | National Census 2020^b^ |  |
| --- | --- | --- | --- | --- | --- | --- | --- | --- |
|  | N | % | 95% CI | % | 95% CI |  | % |  |
| **Male** | **2,642** | **47.48** | **(46.17**–**48.80)** | **47.48** | **(45.06**–**49.91)** |  | **51.00%** |  |
| Age 16–19 | 158 |  |  |  |  |  |  |  |
| High school, technical school | 123 | 77.85 | (70.76–83.62) | 97.08 | (95.42–98.73) |  | 80.95% |  |
| College, university, or above | 35 | 22.15 | (16.38–29.24) | 2.92 | (1.27–4.58) |  | 19.05% |  |
| Age 20–24 | 715 |  |  |  |  |  |  |  |
| High school, technical school | 203 | 28.39 | (25.21–31.81) | 67.32 | (62.37–72.27) |  | 52.27% |  |
| College, university, or above | 512 | 71.61 | (68.19–74.79) | 32.68 | (27.73–37.63) |  | 47.73% |  |
| Age 25–29 | 1,769 |  |  |  |  |  |  |  |
| High school, technical school | 525 | 29.68 | (27.59–31.85) | 68.72 | (65.61–71.83) |  | 60.51% |  |
| College, university, or above | 1,244 | 70.32 | (68.15–72.41) | 31.28 | (28.17–34.39) |  | 39.49% |  |
| **Female** | **2,922** | **52.52** | **(51.20– 53.83)** | **52.52** | **(50.09**–**54.94)** |  | **49.00%** |  |
| Age 16–19 | 363 |  |  |  |  |  |  |  |
| High school, technical school | 247 | 68.04 | (63.08–72.63) | 95.56 | (94.24–96.87) |  | 81.43% |  |
| College, university, or above | 116 | 31.96 | (27.37–36.92) | 4.44 | (3.13–5.76) |  | 18.57% |  |
| Age 20–24 | 902 |  |  |  |  |  |  | |
| High school, technical school | 344 | 38.14 | (35.02–41.35) | 70.79 | (67.05–74.53) |  | 55.78% | |
| College, university, or above | 558 | 61.86 | (58.65– 64.98) | 29.21 | (25.47–32.95) |  | 44.22% | |
| Age 25–29 | 1,657 |  |  |  |  |  |  | |
| High school, technical school | 685 | 41.34 | (38.99–43.73) | 72.18 | (69.52–74.84) |  | 63.26% | |
| College, university, or above | 972 | 58.66 | (56.27–61.01) | 27.82 | (25.16–30.48) |  | 36.74% | |

CI: Confidence interval

a. Weighted to be nationally representative based on the 2019 Comprehensive Survey of Living Conditions.
b. National Census 2020 data were sourced from the Statistics Bureau of Japan.

Supplementary Table S2. Prevalence of Any Lifetime Waterpipe Use by Age Group

|  |  | n/N (%)^a^ | | | |
| --- | --- | --- | --- | --- | --- |
| Age |  |  |  | Sex | |
|  |  | Total  (N = 29,268) |  | Male (N = 14,452) | Female (N = 14,816) |
| 16–19 |  | 28/521 (5.4) |  | 14/158 (8.9) | 14/363 (3.9) |
| 20–24 |  | 213/1,617 (13.2) |  | 120/715 (16.8) | 93/902 (10.3) |
| 25–29 |  | 506/3,426 (14.8) |  | 323/1,769 (18.3) | 183/1,657 (11.0) |
| 30–34 |  | 224/2,185 (10.3) |  | 142/1,019 (13.9) | 82/1,166 (7.0) |
| 35–39 |  | 226/2,640 (8.6) |  | 146/1,338 (10.9) | 80/1,302 (6.1) |
| 40–44 |  | 172/2,455 (7.0) |  | 116/1,251 (9.3) | 56/1,204 (4.7) |
| 45–49 |  | 124/2,582 (4.8) |  | 80/1,283 (6.2) | 44/1,299 (3.4) |
| 50–54 |  | 80/2,503 (3.2) |  | 64/1,261 (5.1) | 16/1,242 (1.3) |
| 55–59 |  | 70/2,118 (3.3) |  | 48/1,084 (4.4) | 22/1,034 (2.1) |
| 60–64 |  | 51/2,276 (2.2) |  | 39/1,123 (3.5) | 12/1,153 (1.0) |
| 65–69 |  | 44/2,209 (2.0) |  | 36/1,108 (3.2) | 8/1,101 (0.7) |
| 70+ |  | 44/4,736 (1.0) |  | 35/2,343 (1.5) | 9/2,393 (0.4) |

a. n/N (%): n = number of waterpipe users; N = total number of individuals in the group; % = proportion of any lifetime waterpipe use within that group.

Supplementary Table S3. Comparison of Characteristics by Waterpipe Use Status Among Teens and Young Adults (Age 16–29)

| Characteristics | N (%)^a^ or *Mean (SD)^b^* | | | | | |  | Comparison^d^ |
| --- | --- | --- | --- | --- | --- | --- | --- | --- |
|  | Total^c^ |  | Never | Ever | Occasional | Regular |  | Crude OR  (95% CI) |
|  | (N = 5,564) |  | 4,817 (86.6) | 391 (7.0) | 252 (4.5) | 104 (1.87) |  |  |
| Age | *24.91 (3.4)* |  | *24.83 (3.4)* | *25.69 (2.7)* | *25.28 (3.1)* | *24.86 (2.9)* |  | 1.06 (1.03–1.08) |
| Sex |  |  |  |  |  |  |  |  |
| Male | 2,642 (47.5) |  | 2,185 (82.7) | 231 (8.7) | 160 (6.1) | 66 (2.5) |  | 1 |
| Female | 2,922 (52.5) |  | 2,632 (90.1) | 160 (5.5) | 92 (3.1) | 38 (1.3) |  | 0.53 (0.45–0.62) |
| Densely Inhabited District (DID) | |  |  |  |  |  |  |  |
| Metropolitan areas | 2,748 (49.4) |  | 2,359 (85.8) | 205 (7.5) | 129 (4.7) | 55 (2.0) |  | 1 |
| Large cities | 806 (14.5) |  | 709 (88.0) | 47 (5.8) | 41 (5.1) | 9 (1.1) |  | 0.83 (0.65–1.05) |
| Accessible small towns | 264 (4.7) |  | 237 (89.8) | 9 (3.4) | 11 (4.2) | 7 (2.7) |  | 0.69 (0.46–1.04) |
| Remote small towns | 278 (5.0) |  | 245 (88.1) | 18 (6.5) | 11 (4.0) | 4 (1.4) |  | 0.82 (0.56–1.19) |
| Accessible rural settlements | 705 (12.7) |  | 606 (86.0) | 51 (7.2) | 33 (4.7) | 15 (2.1) |  | 0.99 (0.78–1.26) |
| Remote rural settlements | 567 (10.2) |  | 493 (86.9) | 47 (8.3) | 19 (3.4) | 8 (1.4) |  | 0.91 (0.70–1.19) |
| Equivalent Household Income | |  |  |  |  |  |  |  |
| Lowest | 1,099 (19.8) |  | 980 (89.2) | 51 (4.6) | 46 (4.2) | 22 (2.0) |  | 1 |
| Lower-middle | 1,574 (28.3) |  | 1,374 (87.3) | 116 (7.4) | 65 (4.1) | 19 (1.2) |  | 1.20 (0.94–1.53) |
| Upper-middle | 1,334 (24.0) |  | 1,175 (88.1) | 74 (5.5) | 54 (4.0) | 31 (2.3) |  | 1.11 (0.86–1.43) |
| Highest | 1,557 (28.0) |  | 1,288 (82.7) | 150 (9.6) | 87 (5.6) | 32 (2.1) |  | 1.72 (1.36–2.17) |
| Education Level | |  |  |  |  |  |  |  |
| High school, technical school | 2,127 (38.2) |  | 1,888 (88.8) | 125 (5.9) | 79 (3.7) | 35 (1.6) |  | 1 |
| College, university, or above | 3,437 (61.8) |  | 2,929 (85.2) | 266 (7.7) | 173 (5.0) | 69 (2.0) |  | 1.20 (0.91–1.59) |
| Other Tobacco Product | |  |  |  |  |  |  |  |
| Conventional cigarette smokers | 618 (11.1) |  | 222 (35.9) | 54 (8.7) | 158 (25.6) | 77 (12.5) |  | 23.4 (19.2–28.4) |
| Conventional cigarette non-smokers | 4,946 (88.9) |  | 4,595 (92.9) | 27 (0.5) | 94 (1.9) | 27 (0.5) |  | 1 |
| HTPs users | 352 (6.3) |  | 66 (18.8) | 110 (31.2) | 119 (33.8) | 57 (16.2) |  | 44.7 (33.6–59.3) |
| HTPs non-users | 5,212 (93.7) |  | 4,751 (91.2) | 281 (5.4) | 133 (2.6) | 47 (0.9) |  | 1 |
| Alcohol Consumption | |  |  |  |  |  |  |  |
| No drinking | 2,208 (39.7) |  | 2,076 (94.0) | 70 (3.2) | 48 (2.2) | 14 (0.6) |  | 1 |
| Light drinking | 2,741 (49.3) |  | 2,313 (84.4) | 223 (8.1) | 141 (5.1) | 64 (2.3) |  | 2.91 (2.37–3.57) |
| Moderate drinking | 548 (9.89) |  | 383 (69.9) | 85 (15.5) | 57 (10.4) | 23 (4.2) |  | 6.78 (5.26–8.73) |
| Heavy drinking | 67 (1.2) |  | 45 (67.2) | 13 (19.4) | 6 (9.0) | 3 (4.5) |  | 7.69 (4.48–13.20) |
| Illicit Drug Usage |  |  |  |  |  |  |  |  |
| No | 5,260 (94.5) |  | 4,707 (89.5) | 324 (6.2) | 166 (3.2) | 63 (1.2) |  | 1 |
| Yes | 304 (5.5) |  | 110 (36.2) | 67 (22.0) | 86 (28.3) | 41 (13.5) |  | 15.00 (11.70–19.30) |
| Social Network |  |  |  |  |  |  |  |  |
| Friends (range: 0–15) | *7.37 (3.9)* |  | *7.18 (3.9)* | *8.72 (3.7)* | *8.50 (3.0)* | *8.69 (3.2)* |  | 1.10 (1.08–1.12) |
| Family (range: 0–15) | *7.70 (3.2)* |  | *7.68 (3.3)* | *7.88 (3.1)* | *7.83 (2.8)* | *8.08 (2.7)* |  | 1.02 (0.99–1.05) |

HTP: Heated Tobacco Product; OR: Odds ratio; CI: Confidence interval; SD: Standard deviation

a. Proportions are within each response group (row percentages).
b. Numbers in *italics* indicate continuous variables and represent mean (SD).
c. Column proportions are shown for the total distribution.
d. Logistic regression analysis was performed. Crude OR compares participants with no lifetime waterpipe use (never users, reference) to those with any lifetime waterpipe use (combining ever, occasional, and regular users).

Supplementary Table S4. Reasons for Smoking Waterpipe in Teens and Young Adults (Age 16–29)

|  | N (%)^a, b^ | | |
| --- | --- | --- | --- |
| Reason | Sex | | Total |
|  | Male  (N = 457) | Female  (N = 290) | (N = 747) |
| **Social Influence** |  |  |  |
| Peer influence | 281 (61.5)* | 186 (64.1)* | 467 (62.5) |
| Family influence | 160 (35.0)*** | 58 (20.0)*** | 218 (29.2) |
| Seen as “cool” | 199 (43.5)*** | 86 (29.7)*** | 285 (38.2) |
| **Product Appeal** |  |  |  |
| Interest in flavors | 212 (46.4)*** | 145 (50.0)*** | 357 (47.8) |
| Perceived less harmful | 204 (44.6)*** | 119 (41.0)*** | 323 (43.2) |
| To avoid bothering others | 154 (33.7)*** | 61 (21.0)*** | 215 (28.8) |
| Interest in shisha bars/cafés | 260 (56.9)*** | 145 (50.0)*** | 405 (54.2) |
| **Smoking Substitution** |  |  |  |
| Where other tobacco is restricted | 168 (36.8)*** | 53 (18.3)*** | 221 (29.6) |
| To quit smoking | 160 (35.0)*** | 60 (20.7)*** | 220 (29.5) |
| To reduce smoking | 150 (32.8)*** | 51 (17.6)*** | 201 (26.9) |

a. Percentages represent column percentages of participants agreeing with the reason.
b. Significance based on Fisher’s Exact Test comparing subgroups (Male vs Female): *p<0.05, **p<0.01, ***p<0.001.

Supplementary Table S5. Knowledge and Attitudes Toward Waterpipe by User Status Among Teens and Young Adults (Age 16–29)

|  | N (%) | | | |  | Comparison^a^ | |
| --- | --- | --- | --- | --- | --- | --- | --- |
|  | Total  (N = 5,564) |  | Never used　waterpipe  (N = 4,817) | Used  waterpipe  (N = 747) |  | Crude OR  (95% CI) | Adjusted OR  (95% CI) |
| Waterpipes are harmful to users | |  |  |  |  |  |  |
| No | 950 (17.1) |  | 677 (71.3) | 273 (28.7) |  | 2.97 (2.50–3.53) | 2.33 (1.86–2.90) |
| Yes | 3,627 (65.2) |  | 3,193 (88.0) | 434 (12.0) |  | 1 | 1 |
| Waterpipes are harmful to others | |  |  |  |  |  |  |
| No | 991 (17.8) |  | 702 (70.8) | 289 (29.2) |  | 3.1 (2.62–3.68) | 2.24 (1.80–2.78) |
| Yes | 3,562 (64.0) |  | 3,145 (88.3) | 417 (11.7) |  | 1 | 1 |
| Waterpipes are “cool” | |  |  |  |  |  |  |
| No | 3,904 (70.2) |  | 3,427 (87.8) | 477 (12.2) |  | 1 | 1 |
| Yes | 685 (12.3) |  | 452 (66.0) | 233 (34.0) |  | 3.7 (3.08–4.46) | 2.62 (2.06–3.33) |

OR: Odds ratio; CI: Confidence interval

a. Logistic regression analysis compares participants with any lifetime waterpipe use (combining ever, occasional, and regular users) against those with no lifetime use (never users, reference). Adjusted OR is controlled for covariates shown in Table 1.

Note: Responses of ‘Unaware of waterpipe’, excluded from OR analyses, constituted 17.7% (harmful to users), 18.2% (harmful to others), and 17.5% (“cool”) of the total sample.

Supplementary Table S6. Weighted Comparison of Characteristics by HTP Use Status Among Teens and Young Adults (Age 16–29)

| Characteristics | N (%)^a^ or *Mean (SD)* | | | | | |  | Comparison^c^ |
| --- | --- | --- | --- | --- | --- | --- | --- | --- |
|  | Total^b^ |  | Never | Ever | Occasional | Regular |  | Crude OR  (95% CI) |
|  | (N = 5,564) |  | 4,342 (78.04) | 372 (6.69) | 246 (4.41) | 605 (10.86) |  |  |
| Age | *24.71 (3.53)* |  | *24.50 (3.6)* | *25.51 (3.0)* | *24.20 (3.7)* | *25.93 (2.6)* |  | 1.09 (1.06–1.11) |
| Sex |  |  |  |  |  |  |  |  |
| Male | 2,642 (47.5) |  | 1,851 (70.1) | 212 (8.0) | 130 (4.9) | 449 (17.0) |  | 1 |
| Female | 2,922 (52.5) |  | 2,491 (85.3) | 160 (5.5) | 115 (3.9) | 156 (5.3) |  | 0.40 (0.35–0.46) |
| Densely Inhabited District (DID) | |  |  |  |  |  |  |  |
| Metropolitan areas | 2,748 (49.4) |  | 2,168 (78.9) | 178 (6.5) | 119 (4.3) | 282 (10.3) |  | 1 |
| Large cities | 784 (14.1) |  | 643 (82.0) | 45 (5.7) | 39 (5.0) | 58 (7.4) |  | 0.82 (0.67–1.01) |
| Accessible small towns | 250 (4.5) |  | 175 (70.1) | 27 (10.7) | 8 (3.0) | 40 (16.1) |  | 1.59 (1.20–2.12) |
| Remote small towns | 306 (5.5) |  | 199 (65.0) | 24 (7.7) | 23 (7.5) | 61 (19.9) |  | 2.02 (1.57–2.60) |
| Accessible rural settlements | 658 (11.8) |  | 500 (75.9) | 35 (5.3) | 33 (5.0) | 91 (13.8) |  | 1.19 (0.97–1.45) |
| Remote rural settlements | 590 (10.6) |  | 467 (79.2) | 49 (8.2) | 15 (2.5) | 60 (10.2) |  | 0.99 (0.79–1.23) |
| Equivalent Household Income | |  |  |  |  |  |  |  |
| Lowest | 1,545 (27.8) |  | 858 (78.1) | 74 (6.7) | 64 (5.8) | 103 (9.4) |  | 1 |
| Lower-middle | 1,323 (23.8) |  | 1,219 (77.4) | 133 (8.4) | 104 (6.6) | 118 (7.5) |  | 1.17 (0.98–1.40) |
| Upper-middle | 1,549 (27.8) |  | 1,038 (77.8) | 102 (7.6) | 67 (5.0) | 127 (9.5) |  | 1.21 (1.02–1.43) |
| Highest | 1,147 (20.6) |  | 1,142 (73.3) | 145 (9.3) | 72 (4.6) | 198 (12.7) |  | 1.16 (0.97–1.40) |
| Education Level | |  |  |  |  |  |  |  |
| High school, technical school | 4,030 (72.4) |  | 3,147 (78.1) | 250 (6.2) | 170 (4.2) | 463 (11.5) |  | 1 |
| College, university or above | 1,534 (27.6) |  | 1,195 (77.9) | 122 (7.9) | 76 (4.9) | 141 (9.2) |  | 1.01 (0.88–1.16) |
| Other Tobacco Product^d^ | |  |  |  |  |  |  |  |
| Conventional cigarette | 519 (9.3) |  | 30 (5.8) | 137 (26.4) | 111 (21.4) | 241 (46.5) |  | 95.20 (65.40–139.00) |
| Waterpipe | 567 (10.2) |  | 109 (19.3) | 135 (23.9) | 125 (22.0) | 198 (34.9) |  | 23.20 (18.60–28.90) |
| Alcohol Usage | |  |  |  |  |  |  |  |
| No drinking | 2,545 (45.8) |  | 2,260 (88.8) | 118 (4.7) | 60 (2.4) | 107 (4.2) |  | 1 |
| Light drinking | 2,484 (44.6) |  | 1,776 (71.5) | 194 (7.8) | 133 (5.4) | 381 (15.3) |  | 3.15 (2.71–3.67) |
| Moderate drinking | 485 (8.7) |  | 269 (55.4) | 57 (11.8) | 49 (10.0) | 110 (22.8) |  | 6.38 (5.13–7.93) |
| Heavy drinking | 50 (0.9) |  | 37 (74.7) | 3 (5.8) | 4 (7.5) | 6 (12.1) |  | 2.68 (1.40–5.15) |
| Illicit Drug Usage |  |  |  |  |  |  |  |  |
| No | 5,348 (96.1) |  | 4,311 (80.6) | 333 (6.2) | 204 (3.8) | 500 (9.4) |  | 1 |
| Yes | 216 (3.9) |  | 31 (14.2) | 40 (18.3) | 42 (19.4) | 104 (48.1) |  | 25.20 (17.10–37.10) |
| Social Network |  |  |  |  |  |  |  |  |
| Friends (range: 0-15) | *6.96 (3.86)* |  | *6.77 (3.9)* | *7.77 (3.7)* | *8.47 (3.5)* | *7.22 (3.6)* |  | 1.06 (1.04–1.08) |
| Family (range: 0-15) | *7.92 (3.29)* |  | *7.90 (3.3)* | *8.22 (3.3)* | *8.50 (3.2)* | *7.67 (3.2)* |  | 1.01 (0.99–1.03) |

HTP: Heated Tobacco Product; OR: Odds ratio; CI: Confidence interval; SD: Standard deviation

Weighted to be nationally representative based on the 2019 Comprehensive Survey of Living Conditions.

a. Proportions are within each response group (row percentages).
b. Column proportions are shown for the total distribution.
c. Logistic regression analysis was performed. Crude OR compares participants with no lifetime HTP use (never users, reference) against those with any lifetime HTP use (combining ever, occasional, and regular users).
d. For other tobacco product rows, crude OR compares users and non-users (reference).

Supplementary Table S7. Weighted Reasons for Smoking HTPs in Teens and Young Adults (Age 16–29)

|  | N (%)^a, b^ | | |
| --- | --- | --- | --- |
| Reason | Sex | | Total |
|  | Male  (N = 791) | Female  (N = 431) | (N = 1,222) |
| **Social Influence** |  |  |  |
| Peer influence | 466 (58.9) | 269 (62.4) | 772 (59.1) |
| Family influence | 337 (42.6) | 167 (38.7) | 506 (38.7) |
| Seen as “cool” | 248 (31.4) | 107 (24.8) | 397 (30.4) |
| **Product Appeal** |  |  |  |
| Interest in flavors | 310 (39.2) | 160 (37.1) | 504 (38.6) |
| Perceived less harmful | 368 (46.5) | 192 (44.5) | 571 (43.7) |
| To avoid bothering others | 337 (42.6)*** | 107 (24.8)*** | 508 (38.9) |
| **Smoking Substitution** |  |  |  |
| Where other tobacco is restricted | 359 (45.4)** | 129 (29.9)** | 498 (38.1) |
| To quit smoking | 280 (35.4)* | 111 (25.8)* | 416 (31.8) |
| To reduce smoking | 325 (41.1)** | 120 (27.8)** | 463 (35.4) |

HTP: Heated Tobacco Product

a. Weighted to be nationally representative based on the 2019 Comprehensive Survey of Living Conditions. Percentages represent column percentages of participants agreeing with the reason.
b. Significance based on Rao-Scott chi-squared test comparing subgroups (Male vs Female): *p<0.05, **p<0.01, ***p<0.001.

Supplementary Table S8. Weighted Knowledge and Attitudes Toward HTPs by User Status Among Teens and Young Adults (Age 16–29)

|  | N (%) | | | |  | Comparison^a^ | |
| --- | --- | --- | --- | --- | --- | --- | --- |
|  | Total  (N = 5,564) |  | Never used　HTPs  (N = 4,257) | Used  HTPs  (N = 1,307) |  | Crude OR  (95% CI) | Adjusted OR  (95% CI) |
| HTP is harmful to users | |  |  |  |  |  |  |
| No | 680 (12.2) |  | 569 (17.3) | 369 (33.5) |  | 4.00 (3.38–4.74) | 2.15 (1.70–2.72) |
| Yes | 3,982 (71.6) |  | 2,715 (82.7) | 735 (66.5) |  | 1 | 1 |
| HTP is harmful to others | |  |  |  |  |  |  |
| No | 722 (13.0) |  | 522 (15.9) | 379 (34.7) |  | 2.60 (2.20–3.07) | 1.79 (1.42–2.25) |
| Yes | 3,908 (70.2) |  | 2,759 (84.1) | 712 (65.3) |  | 1 | 1 |
| HTP is “cool” | |  |  |  |  |  |  |
| No | 4,168 (74.9) |  | 2,963 (90.1) | 749 (67.9) |  | 1 | 1 |
| Yes | 524 (9.4) |  | 327 (9.9) | 355 (32.1) |  | 6.79 (5.60–8.24) | 4.49 (3.46–5.82) |

HTP: Heated Tobacco Product; OR: Odds ratio; CI: Confidence interval

Weighted to be nationally representative based on the 2019 Comprehensive Survey of Living Conditions.

a. Logistic regression analysis compares participants with any lifetime HTP use (combining ever, occasional, and regular users) against those with no lifetime HTP use (never users, reference). Adjusted OR is controlled for covariates shown in Table 1.

Note: Responses of ‘Unaware of waterpipe’, excluded from OR analyses, constituted 13.5% (harmful to users), 13.7% (harmful to others), and 13.3% (“cool”) of the total sample.
